# Surgical Solution of C-Section Associated Surgical Site Infection in LMICs: A Narrative Review

**DOI:** 10.12669/pjms.42.(11AASC).15678

**Published:** 2026-04

**Authors:** Shaikh Habiba Ahmed, Zaheena Shamsul Islam, Syeda Mahrukh Zaidi, Tehreem Khalid

**Affiliations:** 1 Dr. Shaikh Habiba Ahmed, MBBS. Department of Obstetrics and Gynecology, Aga Khan University Hospital, Karachi, Pakistan; 2 Dr. Zaheena Shamsul Islam, MBBS, FCPS, MRCOG. Department of Obstetrics and Gynecology, Aga Khan University Hospital, Karachi, Pakistan; 3 Dr. Mahrukh Fatima Zaidi, MBBS. Department of Obstetrics and Gynecology, Aga Khan University Hospital, Karachi, Pakistan; 4 Dr. Tehreem Khalid, MBBS. Department of Obstetrics and Gynecology, Aga Khan University Hospital, Karachi, Pakistan

**Keywords:** Cesarean section, Dual Antibiotic Prophylaxis, LMICs, LMIC Checklist, Infection Prevention, Surgical Site Infection, Surgical Management

## Abstract

Cesarean section (CS) is a life-saving procedure worldwide, accompanied by a substantial burden of surgical site infections (SSIs), with the most significant impact felt in low- and middle-income countries (LMICs). This review consolidates current knowledge on the surgical management of post-cesarean infections, highlighting the vaginal microbiome as a key source of infection and the physiological immunosuppression in the postpartum period. We describe the categories of deep and organ-space SSIs that require operative management, from wound debridement to laparoscopic drainage and laparotomy. The analysis also assesses proven preventive measures, such as dual-agent antibiotic prophylaxis, advanced closure techniques, and structured care bundles. It includes a standardized, context-sensitive checklist designed for LMICs, which combines pre, intra, and postoperative measures to prevent and manage SSIs, with the aim to reduce maternal morbidity and mortality in resource-limited settings.

## INTRODUCTION

The cesarean section (CS) rate has exponentially increased around the world, and now over a fifth (over 21%) of all pregnant women are delivered by the abdominal route.^1^ Although CS is a critical life-saving procedure, it is still associated with a markedly higher risk of postoperative complications than vaginal birth, with surgical site infection being the most common cause of morbidity.^2^ The incidence of CS-SSIs is disproportionately high in LMICs like Pakistan, with an incidence reported to be 5 to 18 times greater than that in high-income countries. This difference is driven by a combination of limited resources^3^,^4^, overcrowded facilities, and suboptimal compliance with infection prevention protocols.^5^

The underlying reasons for this very high CS-associated SSI are the immunological adaptations of pregnancy, vaginal microbiota, and nutritional deficiencies.^2^,^3^,^6^, This review aims to detail the surgical approaches to CS-SSIs, paying special attention to the vaginal origin of pathogens, the altered maternal immune status, and a practical standard of care management guideline for low-resource settings. The objective was to provide a comprehensive review for addressing this major cause of maternal illness.

### Factors resulting in high burden of cs-associated surgical site infection in LMICs:

The increasing frequency of CS in LMICs does not consistently correlate with better patient outcomes, with a wide disparity between the rich and poor. In numerous instances, the rise of CS is attributable to non-clinical factors such as patient preference, cultural and social reasons, access to healthcare facilities, financial and logistic concerns, fear of litigation, as well as obstetric factors such as parity, comorbidities, previous C-section, number of fetuses, and fetal life. In several low- and middle-income countries, although the total risk is increasing, the proportion of birth by CS in vulnerable pregnant women is zero percent due to the non-accessibility of this life-saving surgery, occurring without concurrent enhancements in surgical safety.^7^ Consequently, a growing number of women undergo surgery in settings that are poorly prepared to prevent complications, resulting in serious morbidity and mortality.

Hygiene deficiencies persist as a major obstacle in many operating theatres within LMICs. Inadequate instrument sterilization, poor handwashing compliance, insufficient environmental decontamination, and the inappropriate reuse of disposable items are all documented issues that directly introduce pathogens to the surgical site.^8^,^9^ Moreover, overcrowding and staffing shortages often result in hurried procedures and lapses in aseptic technique, creating ideal conditions for SSI development. These systemic weaknesses highlight that addressing CS-SSIs demands not just clinical measures but also a fundamental reinforcement of health system infrastructure and strict adherence to IPC fundamentals.^10^

### Vaginal Microbiome:

The cesarean section incision is made in close anatomical relation to the cervix and vaginal canal. Even with strict aseptic protocols, the procedure inherently carries a risk of contaminating the abdominal wound with cervico-vaginal flora. The vagina hosts a complex microbial community, and under specific conditions, its resident bacteria can initiate infection.^11^ Polymicrobial infections are typical, often involving *Escherichia coli*, *Enterococcus faecalis*, anaerobic species like *Bacteroides*, and Group-B *Streptococcus*.^12^,^13^ Although *Staphylococcus aureus* from the skin is a leading pathogen, the significant presence of gram-negative and anaerobic bacteria points to the genital tract’s role.^13^,^14^ Clinical factors such as prolonged labor, premature rupture of membranes, and repeated vaginal exams increase the bacterial burden and disrupt normal flora, thereby raising the risk of wound contamination.^6^ Dysbiosis, including bacterial vaginosis, is a recognized independent risk factor for post-cesarean infections like endometritis, supporting the use of antibiotic prophylaxis that includes anaerobic coverage.^15^

### Altered Maternal Immunity:

Pregnancy induces a unique immunological state tailored to tolerate the semi-allogeneic fetus, characterized by a shift toward anti-inflammatory and regulatory pathways.^3^ While vital for gestation, this adaptation can simultaneously increase vulnerability to certain pathogens. Following the physiological stress of surgery, this modified immune response may be insufficient to mount the robust inflammation needed to prevent surgical site infections. Several comorbid conditions common in LMICs can further impair immune function and significantly elevate SSI risk.^16^

- Anemia, highly prevalent in these regions, compromises oxygen delivery and leukocyte activity, critically undermining wound healing and bacterial clearance.^17^
- Malnutrition depletes the protein and micronutrient stores essential for an effective immune response and tissue repair.^3^
- Obesity is a major risk factor, with SSI rates climbing alongside BMI.^18^ Adipose tissue is relatively hypoxic, and its poor blood supply hinders immune cell migration and antibiotic penetration.^19^
- Diabetes and Hyperglycemia: Inadequate perioperative glucose control is strongly linked to impaired wound healing and infection. A key management goal is maintaining postoperative blood glucose below 180-200 mg/dL.^20^

Therefore, managing SSIs requires an appreciation of this inherently susceptible host state, where infections can advance more quickly and with increased severity.

### Classification of SSI:

Precise SSI classification is essential for guiding management, especially in deciding when an operation is needed.^21^

### • Superficial Incisional SSI:

Involves only skin and subcutaneous tissue.

### • Deep Incisional SSI:

Involves the deep soft tissues (fascia and muscle). This category often manifests as fascial separation.^22^

### • Organ/Space SSI

Involves any anatomical structure deeper than the fascia that was handled during the operation (e.g., endometritis, pelvic abscess).^21^

From a surgical perspective, deep incisional and organ/space infections present the most serious challenges and demand a high level of clinical suspicion.

### From medical therapy to surgical exploration - A comparison:

### Initial Assessment and Triage:

The assessment begins with rapid triage and prioritizing hemodynamic stability. Broad-spectrum intravenous antibiotics covering Gram-positive, Gram-negative, and anaerobic bacteria should be initiated without delay, with subsequent refinement guided by culture data. The Laboratory Risk Indicator for Necrotizing Fasciitis Score (LRINEC) may act as an adjunct; laboratory studies such as complete blood count and septic blood profile are essential. Transvaginal and transabdominal ultrasound is the initial modality for evaluating pelvic collections, while contrast-enhanced CT – Abdomen and Pelvis is needed for defining complex, multiloculated abscesses and planning an intervention strategy. The choice between percutaneous drainage, wound debridement, laparoscopic intervention, and exploratory laparotomy hinges on the characteristics of the collection.^23^

### Minimally Invasive Drainage Techniques:

Image-guided drainage via transvaginal, transrectal, or transabdominal routes serves as a first-line, minimally invasive intervention for many pelvic abscesses. Success rates are high, often exceeding 90% in suitable cases, for symptomatic, well-defined pelvic collections. Selection of a short, safe route to avoid bowel and bladder, use of real-time ultrasound, choice of catheter (8-14Fr), including regular flushing and removal upon cavity resolution, brings out the ideal outcome. However, this approach is often inadequate for multiloculated and phlegmonous collections or dense adhesions, which usually require operative management.^24^

### Management of Post-Caesarean Fluid Collections-Hematoma and Seroma:

### Incidence and Etiology:

Hematomas and seromas most frequently originate in the subcutaneous tissue plane, particularly in cases where the tissue depth exceeds 2 cm or when surgical haemostasis is incomplete. The persistence of this “dead space” facilitates the accumulation of blood or serous fluid. Epidemiological data from various clinical settings, particularly in low and middle-income countries (LMICs), indicate a reported incidence ranging from 3% to 15% within the initial postoperative week.^25^ They act as a potent risk factor for subsequent surgical site infection (SSI) by providing a medium for bacterial proliferation and impairing efficient wound healing.

### A Stratified Approach for Management:

The management of post-cesarean fluid collections is guided by collection type and clinical presentation. Asymptomatic hematomas may resolve spontaneously, but symptomatic collections generally require formal evacuation. This involves sterile aspiration, clot removal, and meticulous hemostasis, with reclosure over a drain if persistent oozing is anticipated.^25^ Seromas are managed through serial needle aspiration every 24-48 hours combined with compression dressings to prevent re accumulation. Persistent or symptomatic seromas beyond the first postoperative week typically warrant surgical re-exploration, especially with signs of inflammation and infection.

Several factors significantly increase wound morbidity risk. Thornburg reported that vertical skin incisions elevate complication risk more than sevenfold, while obesity independently increases both infection (OR 5.16; 95% CI 2.3–11.8) and wound dehiscence (OR 10.7; 95% CI 4.0–29.2) risks across all severity levels.^26^ Regional studies note higher seroma incidence following emergency cesareans, potentially related to compromised hemostasis under time constraints, elevated BMI, and reduced surgical duration, though multicenter validation remains lacking.

### Management of Post-Caesarean Wound Infection:

### Choice of antibiotic:

Treatment selection for post-cesarean wound infections depends on clinical presentation. Non-purulent cellulitis typically responds to oral antibiotics covering β-hemolytic streptococci and MSSA, including cephalexin, cefadroxil, or dicloxacillin. The emergence of purulent drainage necessitates expanded coverage for MRSA using agents such as clindamycin, trimethoprim-sulfamethoxazole, or doxycycline.^27^ This approach is particularly crucial in South Asian contexts, where MRSA prevalence reaches 47.7% in obstetric wound isolates, supporting empiric MRSA coverage when drainage is evident.^28^

### Incision and Drainage (I&D):

Wounds demonstrating purulent drainage, fluctuance, or separation require formal incision and drainage. This involves complete wound exploration with disruption of loculations and excision of necrotic tissue until viable bleeding edges are visualized. Fascial integrity must be confirmed during this procedure, as dehiscence creates a surgical emergency requiring immediate operative repair.^29^

### Wound Management Techniques:

Following I&D, deep wounds typically require packing with saline-moistened gauze changed twice daily. These dressings function through osmotic action: as saline evaporates, the hypertonic environment draws fluid from wound tissues. However, protein deposition from wound exudate can create an impermeable barrier, necessitating frequent dressing changes to maintain therapeutic effectiveness.^30^ While advanced wound care materials (foam, alginates, hydrocolloids) are present, systematic reviews demonstrate equivalent healing rates with traditional gauze, making it a practical choice in resource-limited settings.^31^

### Definitive Closure Options:

Delayed primary closure (DPC) provides an optimal balance between infection control and healing efficiency for contaminated wounds. Performed after establishing clean granulation tissue, DPC achieves 81-100% success rates in observational studies, reducing healing time to 2-3 weeks compared to 6-10 weeks with secondary intention.^32^ Failure typically results from incomplete debridement or undrained abscess collections.

### Necrotizing Fasciitis After Cesarean Delivery:

post-cesarean necrotizing fasciitis, although rare (<0.5%), carries extremely high morbidity and mortality and is reported more frequently in high-risk populations, particularly those with poorly controlled diabetes, anemia, or immunosuppression. Management requires urgent and aggressive surgical debridement, often with repeat operations every 24–36 hours until all necrotic tissue is removed. Empiric antibiotics must provide broad coverage, typically including agents against MRSA (Vancomycin/Linezolid/Daptomycin), Gram-negative, and anaerobic organisms (Piperacillin-tazobactam or Carbapenem or Ceftriaxone + Metronidazole). Once Group-A streptococcal infection is confirmed, therapy can be narrowed to Penicillin + Clindamycin. Evidence for adjunctive IVIG remains mixed, and its benefit in post-cesarean necrotizing infections is not clearly established.^23^

**Table-I:** LMIC Checklist for Prevention and Management of CS-SSIs

Phase	Intervention	Key Action
Pre-operative	1. Patient Optimization	Assess and correct severe anemia (Hb <7g/dL) to optimize preoperative health.^17^
	2. Vaginal Preparation	Cleanse vagina with povidone-iodine solution to reduce bacterial load from primary source.^41^
	3. Antibiotic Prophylaxis	Give IV Cefazolin within 60 min pre-incision. Add IV Metronidazole for high-risk cases (in labor, ROM) to cover skin and vaginal flora, including anaerobes.^34^
	4. Skin Antisepsis	Prep abdomen with 2% chlorhexidine in alcohol for reducing skin bacterial counts.^17^
Intra-operative	5. Aseptic Technique	Strictly follow the WHO Surgical Safety Checklist. Enhances teamwork and aseptic adherence.^2^
	6. Surgical Technique & Closure	Meticulous hemostasis, gentle handling. Close subcutaneous tissue if ≥2 cm. Consider antimicrobial sutures if available. Maintain normothermia, reduce dead space, bacterial colonization, and immune suppression.^20^,^35^
Post-operative	7. Wound Care & Dressings	First inspection at 48 hours. Consider silver or iNPWT dressings for high-risk patients (e.g., obese, diabetic). It enables early SSI detection and provides antimicrobial protection.^38^,^39^
	8. Surveillance & Education	Teach patient SSI warning signs. Arrange follow-up. Catches post-discharge SSIs.^21^
Management	9. Diagnosis	For deep/organ-space SSI: Start broad-spectrum IV antibiotics. Use Ultrasound/CT if abscess suspected.
	10. Surgical Intervention	Deep SSI: Surgical debridement. Pelvic Abscess: Attempt image-guided drainage^49^; if unsuccessful, proceed to laparoscopy/laparotomy. Necrotizing Fasciitis: Immediate radical debridement^50^.

### Post-Cesarean Endometritis:

Endometritis is among the most common infectious complications after cesarean delivery, typically presenting within 48–72 hours with fever, uterine tenderness, and foul lochia. The recommended regimen supported by decades of clinical experience is IV Clindamycin plus Gentamicin. Extended-interval gentamicin (5 mg/kg/day) dosing is equally effective and more cost-efficient, making it particularly useful in high-volume, resource-limited wards.^23^ Ampicillin is added if enterococcus is suspected. If fevers persist beyond 48–72 hours despite appropriate antibiotics, clinicians should evaluate for deep pelvic abscess, infected hematoma, or retained products.

### Surgical Management of Severe or Refractory Post-Cesarean Infections:

When post-cesarean infections progress despite conventional treatment, exploratory laparotomy provides definitive intervention. Specific indications include complex multiloculated abscesses inaccessible to percutaneous drainage, uterine wound dehiscence with tissue necrosis, and necrotizing infections involving deeper fascial planes. Clinical deterioration during appropriate antibiotic therapy represents another critical indication for surgical exploration.

The procedure’s substantial risks are highlighted by recent meta-analytical data demonstrating a 7.24% maternal mortality rate following relaparotomy, with significant disparities between healthcare settings (16.9% in low-resource versus 0.56% in high-resource environments).^32^ Surgical management requires comprehensive abdominal exploration, complete drainage of purulent collections, and excision of non-viable tissue. While uterine conservation may be possible with limited dehiscence and viable tissue, hysterectomy becomes necessary with extensive necrosis or hemorrhage. Planned Re-exploration (“second-look” laparotomy) within 24-48 hours is standard in severe cases to ensure adequate source control.

Despite its potential benefits, the procedure carries significant morbidity, including prolonged ileus, adhesion formation, and surgical site re-infection. The decision to proceed with laparotomy therefore requires careful assessment of risks against the potentially life-threatening consequences of uncontrolled infection.

### Evidence-Based Prevention Strategies:

SSI prevention is far more efficient and cost-effective than treatment.

- ***Antibiotic Prophylaxis:*** This is the single most important preventive measure. The standard is a single weight-based dose of a first-generation cephalosporin (e.g., cefazolin) given IV within 60 minutes before incision.^33^ Considering the vaginal pathogen source, dual therapy with cefazolin and metronidazole extends coverage to anaerobic bacteria and is strongly supported by data from hysterectomy studies showing substantial SSI reduction.^34^
- ***Surgical Closure and Suture Selection:*** Meticulous surgical technique during closure is critical. The use of triclosan-coated antimicrobial sutures for fascial and subcutaneous closure has been shown to reduce the risk of microbial colonization and SSI.^35^,^36^ For patients with a subcutaneous fat layer ≥2 cm in depth, closure of this layer is recommended to eliminate dead space, which can serve as a nidus for infection and hematoma formation.^20^
- ***Advanced Wound Dressings:*** The choice of postoperative dressing can influence infection rates, particularly in high-risk patients.
  *- ** Silver-Impregnated Dressings:*** These dressings provide a broad-spectrum antimicrobial barrier at the incision site. A randomized trial demonstrated that silver nylon dressings significantly reduced the risk of superficial SSI after cesarean delivery compared to standard dressings.^37^
  *-** Incisional Negative Pressure Wound Therapy (iNPWT):*** This technology involves applying controlled suction to a closed incision. It helps manage exudate, reduces edema, and may improve perfusion. Prophylactic iNPWT has been shown to significantly reduce SSI risk in high-risk populations, such as obese women undergoing CS.^38^,^39^ While cost can be a barrier in LMICs, low-cost, improvised systems have been successfully used.^40^
- ***The imperative role of vaginal preparation:*** In light of the vagina’s confirmed role as an infection source, preoperative cleansing with an antiseptic is a fundamental preventive step. A Cochrane review and subsequent randomized controlled trials have established that vaginal preparation with povidone-iodine solution significantly lowers the incidence of postoperative endometritis and overall infectious morbidity.^15^,^41^ The ideal antiseptic agent is still under investigation. While povidone-iodine is widely utilized^42^,^43^ chlorhexidine gluconate (CHG) is often favored for its broad-spectrum efficacy and persistent action.^17^,^44^ Crucially, alcohol-based solutions, common for abdominal skin prep, are not suitable for vaginal use due to the potential for mucosal irritation and injury.^45^
- ***Infection Prevention Bundles:*** These are structured sets of evidence-based practices that, when applied collectively, achieve a greater reduction in infection rates than individual measures.^46^
- ***Core elements encompass:***
  ***- Preoperative:*** Patient education, CHG bathing, glycemic control, and vaginal preparation with povidone-iodine.
  ***- Intraoperative:*** Proper antibiotic timing, abdominal prep with chlorhexidine gluconate (CHG)-alcohol, normothermia maintenance, atraumatic technique, and use of antimicrobial sutures where available.
  ***- Postoperative:*** Early dressing removal, glucose management, and post-discharge monitoring. Implementing such bundles has repeatedly been shown to cut SSI rates in gynecologic surgery by more than half.^47^

### Implications for low-resource settings (LMICs):

The severe burden of CS-SSIs in LMICs calls for practical, context-driven solutions. The assembled evidence highlights several critical implications:

### Systemic Foundation:

Tackling unhygienic practices via improved sterilization, hand hygiene, and cleaning is imperative and forms the base for all other interventions.^8^,^10^

### Prioritizing Cost-Effective Prevention:

Inexpensive, high-impact measures like vaginal preparation, appropriate antibiotic prophylaxis, and subcutaneous fat closure must be standardized and prioritized.^16^,^41^

### Building Surgical Capacity:

Developing skills for essential procedures like debridement and, where possible, laparoscopic drainage is vital. Training must focus on recognizing severe infections and the timely use of re-laparotomy.

### Utilizing Checklists:

Standardized checklists, such as the one proposed, can help surmount knowledge-practice gaps and ensure reliable application of evidence-based care in busy, resource-poor environments.^2^

To address the systemic challenges behind the high burden of CS-SSIs in LMICs, a multi-faceted governance and partnership-based approach is essential. Strong national health governance is critical for standardizing, disseminating, and enforcing SSI prevention policies^46^,^47^ where strong regulatory frameworks have proven to enhance compliance. This top-down direction must be synergized with operational public-private partnerships (PPPs), which can directly overcome resource barriers by leveraging private sector capabilities for the transfer of essential resources, trainings aligned with public health guidelines, and sustainable access to sterile equipment in underserved areas. Furthermore, a closed-loop liaison that actively involves government bodies, academic associations, and community leaders is vital for mobilizing funding, driving patient education, and conducting audits and quality control. This integrated strategy, combining regulatory enforcement, strategic resource-sharing via PPPs, and multi-stakeholder collaboration, offers a sustainable and holistic pathway to significantly improve surgical care delivery and maternal and child healthcare.^48^

### A proposed standard of care checklist for LMICS:

Drawing on the evidence, the following checklist offers a practical, sequential guide for LMIC healthcare teams.

## DISCUSSION

This narrative review amalgamates the complex challenges and solutions related to surgical site infections after cesarean sections in LMICs. The evidence shows that CS-SSI arises from interplay of microbial, host and systemic factors with vaginal dysbiosis^15^,^41^ and peripartum immunosuppression,^3^,^17^ worsened by LMIC comorbidities such as anemia, rising rate of obesity and malnutrition.

A key analysis of management options reveals that while antibiotics suffice for superficial infections, the data compellingly show that for deep and organ-space SSIs, prompt surgical intervention is crucial for success.^51^-^54^ Surgical care in LMICs should balance minimally invasive approaches, like laparoscopic drainage for stable abscesses, with life-saving laparotomy for severe cases, ensuring timely intervention to prevent complications in septic patients.^55^-^58^

In LMICs, the most effective CS-SSI prevention relies on low-cost, high-value measures. Dual antibiotic prophylaxis targeting skin flora and vaginal anaerobes, combined with skin and vaginal antisepsis, forms a core strategy.^17^,^34^,^41^ Proper surgical technique, including deep subcutaneous closure and antimicrobial sutures, reduces infection-prone spaces.^20^,^35^ Advanced dressings, such as silver-impregnated materials or iNPWT for high-risk patients, further protect incisions in early postoperative period.^37^,^38^ Bundled interventions can cut SSI rates by over 50%, highlighting the value of standardization and team-based care, while simple actionable checklists ensure consistent application in busy LMIC settings.^2^,^46^,^47^

Ultimately, reducing the CS-SSI burden in LMICs is not just a clinical issue but a health system one. It demands a two-fold commitment: first, to strengthen foundational infrastructure and IPC protocols to eliminate unhygienic practices;^8^ and second, to equip frontline providers with the knowledge, skills, and simple instruments to implement proven preventive and therapeutic measures. By closing this gap between knowledge and practice, significant progress can be made in protecting maternal health.

### Contextual Challenges in Low- and Middle-Income Countries:

- Higher prevalence of anemia, obesity, diabetes, and emergency cesarean delivery contribute to increased wound complications compared to high-income settings.
- Limited access to sterile dressing materials or postoperative follow-up may delay detection of early wound infections.
- Antibiotic resistance patterns (notably MRSA) should be guided by careful consideration when selecting empiric therapy.
- Clinical understanding is currently based on small, single-center studies. There is a critical need for large, prospective, multi-center registries to establish accurate epidemiology and guide effective prevention strategies.

## CONCLUSION

Surgical site infections following cesarean section pose a severe, yet largely preventable, threat to maternal health in LMICs. The increasing CS rate highlights the urgent need for effective infection control measures. A multifaceted strategy is essential, focused on the consistent application of a bundled prevention approach including appropriate antibiotics, skin and vaginal antisepsis, meticulous closure techniques, and careful post-operative care. For established infections, a stepped management philosophy is critical: antibiotics for mild cases, with a low threshold to advance to source control using the entire range of surgical options, from minimally invasive drainage to prompt exploratory laparotomy. By inserting these evidence-based practices within strengthened health systems, the heavy burden of CS-SSIs can be alleviated, thereby saving lives and progressing toward safe motherhood for all.

### Authors Contribution:

**ZSI:** Conceptualize, designed and conducted final review and revision of manuscript, is responsible for the integrity of the research.

**SHA:** Literature search, manuscript writing, drafted the initial manuscript.

**TK & MFZ:** Manuscript writing with specific subject based input.

All authors have read and approved the final manuscript.
